# TFEB controls syncytiotrophoblast formation and hormone production in placenta

**DOI:** 10.1038/s41418-024-01337-y

**Published:** 2024-07-04

**Authors:** Marcella Cesana, Gennaro Tufano, Francesco Panariello, Nicolina Zampelli, Chiara Soldati, Margherita Mutarelli, Sandro Montefusco, Giuseppina Grieco, Lucia Vittoria Sepe, Barbara Rossi, Edoardo Nusco, Giada Rossignoli, Giorgia Panebianco, Fabrizio Merciai, Emanuela Salviati, Eduardo Maria Sommella, Pietro Campiglia, Graziano Martello, Davide Cacchiarelli, Diego Luis Medina, Andrea Ballabio

**Affiliations:** 1 Telethon Institute of Genetics and Medicine (TIGEM), 80078 Pozzuoli, Naples, Italy; 2 Department of Advanced Biomedical Sciences, Federico II University, 80131 Naples, Italy; 3 National Research Council of Italy (CNR), Institute of Applied Sciences and Intelligent Systems “Eduardo Caianiello”, Pozzuoli, Italy; 4 Department of Biology, University of Padua, Padua, Italy; 5 Department of Pharmacy, University of Salerno, Fisciano, 84084 Salerno, Italy; 6 Department of Translational Medical Sciences, Federico II University, 80131 Naples, Italy; 7 SSM School for Advanced Studies, Federico II University, Naples, Italy; 8 Department of Molecular and Human Genetics, Baylor College of Medicine, Houston, TX 77030 USA; 9 Jan and Dan Duncan Neurological Research Institute, Texas Children’s Hospital, Houston, TX 77030 USA

**Keywords:** Molecular biology, Cell biology

## Abstract

TFEB, a bHLH-leucine zipper transcription factor belonging to the MiT/TFE family, globally modulates cell metabolism by regulating autophagy and lysosomal functions. Remarkably, loss of TFEB in mice causes embryonic lethality due to severe defects in placentation associated with aberrant vascularization and resulting hypoxia. However, the molecular mechanism underlying this phenotype has remained elusive. By integrating in vivo analyses with multi-omics approaches and functional assays, we have uncovered an unprecedented function for TFEB in promoting the formation of a functional syncytiotrophoblast in the placenta. Our findings demonstrate that constitutive loss of TFEB in knock-out mice is associated with defective formation of the syncytiotrophoblast layer. Indeed, using in vitro models of syncytialization, we demonstrated that TFEB translocates into the nucleus during syncytiotrophoblast formation and binds to the promoters of crucial placental genes, including genes encoding fusogenic proteins (Syncytin-1 and Syncytin-2) and enzymes involved in steroidogenic pathways, such as CYP19A1, the rate-limiting enzyme for the synthesis of 17β-Estradiol (E2). Conversely, TFEB depletion impairs both syncytial fusion and endocrine properties of syncytiotrophoblast, as demonstrated by a significant decrease in the secretion of placental hormones and E2 production. Notably, restoration of TFEB expression resets syncytiotrophoblast identity. Our findings identify that TFEB controls placental development and function by orchestrating both the transcriptional program underlying trophoblast fusion and the acquisition of endocrine function, which are crucial for the bioenergetic requirements of embryonic development.

## Introduction

Transcription factor EB (TFEB), a member of the microphthalmia family of bHLH-leucine zipper factors [1], plays a crucial role in the modulation of cellular metabolism primarily through transcriptional regulation of lysosomal biogenesis and autophagy [2, 3]. TFEB function is regulated by the Mechanistic Target of Rapamycin Complex 1 (mTORC1) [4–6], which phosphorylates TFEB in a substrate-specific manner resulting in its cytoplasmic retention [7]. Following diverse stimuli, TFEB is dephosphorylated and rapidly translocates into the nucleus to orchestrate a transcriptional program that integrates energy availability with cellular demand [8].

Genetic ablation of TFEB in mice was associated with defects in placental vascularization, resulting in severe hypoxia and embryo death at E10.5 [9]. This is consistent with TFEB’s role in the proliferation of endothelial cells [10]. However, a thorough molecular characterization of TFEB role in placentation has never been performed. The placenta is a transitory organ vital for the health of the fetus and the mother. Its structure undergoes continuous changes throughout gestation thanks to the highly dynamic and specialized nature of the different subtypes of trophoblast populations. In vivo, proliferative villous cytotrophoblasts act as the progenitor milieu of the placental epithelium and differentiate to generate extravillous trophoblast cells (EVT) and syncytiotrophoblast (STB) [11]. EVTs anchor the placenta to the maternal decidua and remodel the uterine spiral arteries to increase the blood flow into the placenta [12, 13]. STB is a multinucleated epithelial layer that lines the placenta villi and mediates the exchange of nutrients and waste and the production of hormones [14], thus orchestrating the maternal physiological adaptation to pregnancy. STB requires regular replenishment throughout pregnancy, provided by differentiation and fusion of cytotrophoblast cells. This process involves profound morphological and biochemical changes. Transcriptional activation of syncytins, which were captured from retroviruses during evolution [15], has proven essential for the generation of syncytia in the placenta by bridging plasma membranes and directly merging cells into multinucleated units [16–18].

A functional and competent STB acts as a full-fledged endocrine organ, secreting protein and steroid hormones into the maternal bloodstream and fetal circulation, including human chorionic gonadotropin (hCG), a key signaling molecule displaying autocrine and paracrine actions, increasing syncytium formation [19] and promoting angiogenesis in uterine endothelium [20]. Also, STB primarily secretes progesterone and estrogens [21, 22], which regulate key processes, such as decidualization of the endometrium, embryo implantation, trophoblast invasion and modulation of maternal vasculature [23]. Regulation of steroidogenesis in the placenta depends on a careful balance between expression and activity of steroidogenic enzymes and availability of steroid precursors. Indeed, alteration of steroid hormone homeostasis has been linked to pregnancy complications, including pre-eclampsia and gestational diabetes [21]. Here, we unveil a yet undescribed function for TFEB in promoting the formation of a functional STB. By integrating in vivo results with multi-omics approaches and functional assays, we found that TFEB orchestrates a transcriptional program that controls STB development and function. Consistently, TFEB depletion impairs STB fusion and function. Notably, we also discovered that TFEB regulates the steroidogenic properties of STB by controlling the expression of CYP19A1, the enzyme responsible for the production of estrogens [24].

## Results

### Syncytiotrophoblast formation is associated with TFEB nuclear translocation

**Fig. 1:**
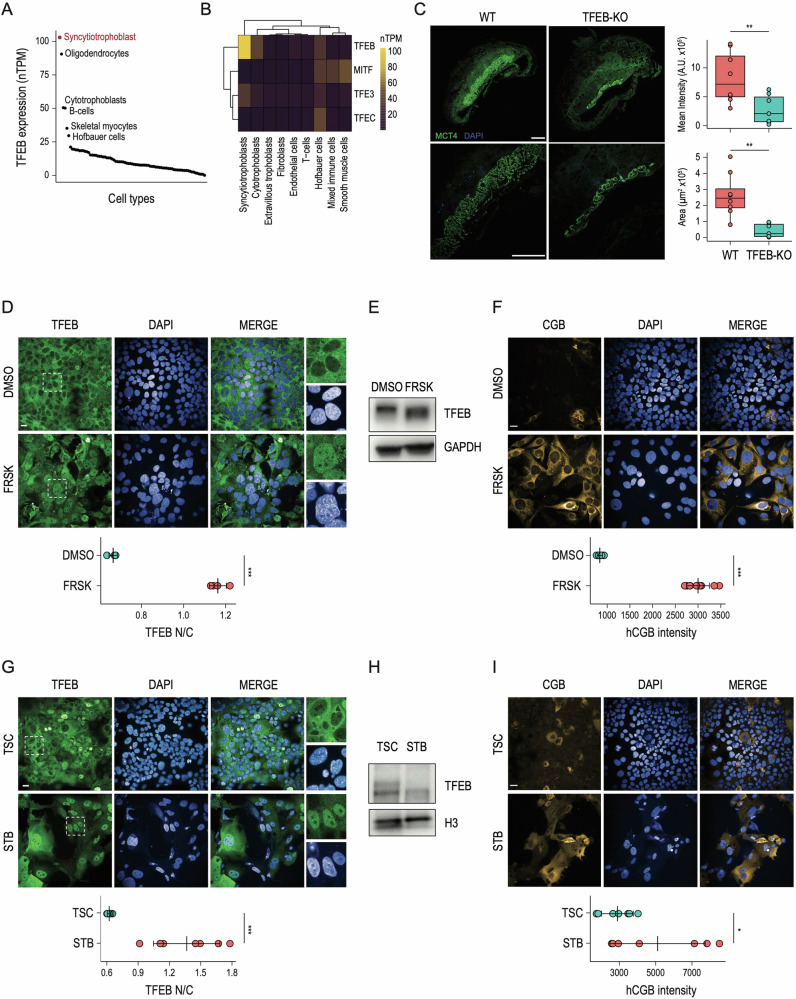
TFEB is activated during in vitro syncytiotrophoblast formation. **A** Dot plot displaying TFEB expression (nTPM) in different cell types according to the Human Protein Atlas single-cell datasets. The top-6 cell types that express the highest levels of TFEB are indicated in black, with the Syncytiotrophoblast representing the cell compartment with TFEB highest levels indicated in red. **B** Heatmap displaying TFEB, TFE3, MITF, and TFEC expression (nTPM) at the single-cell level in human placenta cell types according to Human Protein Atlas clustered single-cell datasets. **C** (Left) E10.5 placentas derived from wild-type (WT) and TFEB knock-out mice (TFEB-KO) were stained for MCT4 and DAPI. Images shown at different magnifications. Scale bars: 500μm (4×) an 500 μm (10×). Sections from three placentas per genotype were analyzed. (Right) Histograms displaying ImageJ-based quantification of MCT4 signal shown as “Mean Intensity” and “Area” (** one-sided p-value < 0.01 with unpaired Welch’s *t*-test). **D** (Up) Representative immunofluorescence images of BeWo cells in DMSO and Forskolin (FRSK) conditions immunostained for TFEB. Nuclei are counterstained blue using DAPI. Scale bar 20 µm. The inset displays a digital magnification of the representative immunofluorence images for a detailed observation of TFEB subcellular localization. (Down) Dot plot showing the ratio relative to the quantification of TFEB fluorescence intensity in the nucleus and the cytoplasm (TFEB N/C) upon indicated treatments (*n* = 4) measured by High-Content Imaging (*** one-sided *p*-value < 0.001 with unpaired Welch’s *t*-test). **E** Representative image of immunoblot analysis of TFEB levels in BeWo cells in DMSO and Forskolin (FRSK) conditions. A molecular weight shift of TFEB is visible in FRSK-treated cells, suggesting that TFEB is dephosphorylated in this condition. GAPDH was used as a loading control. **F** (Up) Representative immunofluorescence images of BeWo cells upon DMSO and Forskolin (FRSK) treatments immunostained for human chorion gonadotropin β subunit (CGB). Nuclei are counterstained blue using DAPI. Scale bar 20 µm. (Down) High-content imaging-based quantification of CGB intensity upon indicated treatments (*n* = 10) shown as dot plots (*** one-sided *p*-value < 0.001 with unpaired Welch’s *t*-test). **G:**(Up) Representative immunofluorescence images of human naive IPSC-derived trophoblast stem cells (TSC) and differentiated syncytiotrophoblast (STB) immunostained for TFEB. Nuclei are counterstained blue using DAPI. Scale bar 20 µm. The inset displays a digital magnification of the representative immunofluorence images for a detailed observation of TFEB subcellular localization. (Down) Dot plot showing the ratio relative to the quantification of TFEB fluorescence intensity in the nucleus and the cytoplasm (TFEB N/C) upon indicated treatments (n = 7) measured by High-Content Imaging (*** one-sided *P* value < 0.001 with unpaired Welch’s *t*-test). **H** Representative image of immunoblot analysis of TFEB levels in human naive IPSC-derived trophoblast stem cells (TSC) and differentiated syncytiotrophoblast (STB). A molecular weight shift of TFEB is visible in STB, suggesting that TFEB is dephosphorylated in this condition. H3 was used as a loading control. **I** (Up) Representative immunofluorescence images of human naive IPSC-derived trophoblast stem cells (TSC) and differentiated syncytiotrophoblast (STB) immunostained for human chorion gonadotropin β subunit (CGB). Nuclei are counterstained blue using DAPI. Scale bar 20 µm. (Down) High-content imaging-based quantification of CGB intensity upon indicated treatments (*n* = 7) shown as dot plots (*** one-sided *p*-value < 0.001 with unpaired Welch’s *t*-test).

We interrogated single-cell published datasets [25] to explore TFEB expression across human tissues and observed that syncytiotrophoblast (STB) represents the site with the highest TFEB levels (Fig. 1A). Also, among all the MiT/TFE family members, TFEB displays the highest expression levels in STB (Fig. 1B). This observation is in line with previous in vivo analyses, showing high TFEB levels in the labyrinthine trophoblasts [9]. Thus, we crossed *Tfeb*+/− heterozygous mice to generate *Tfeb−/−* homozygous knock-out (KO) mice. We collected placenta tissues from wild-type and TFEB KO animals at 10.5 dpc, which is the time point at which embryonic lethality was previously observed [9]. Interestingly, immunofluorence analysis for the monocarboxylate transporters of lactate MCT4 (SLC16A3) located at the syncytiotrophoblast layer II (SynT-II) basal membranes [26] revealed a significant reduction of signal intensity and distribution in placentas from TFEB KO mice compared to wild-type animals (Fig. 1C), suggesting that TFEB is required for SynT-II formation.

Thus, to define the molecular mechanism underlying TFEB’s role in STB formation we leveraged two distinct human cellular systems, which recapitulate the main features of STB biology in vitro. First, we employed BeWo cells, widely used to study syncytialization of trophoblasts following the administration of cAMP analogs, such as Forskolin (FRSK) [27, 28]. Indeed, activation of the cAMP-PKA signaling pathway triggers the intercellular fusion of cytotrophoblast into STB via a modified epithelial-mesenchymal transition characterized by a reorganization of cytoskeletal components and junctional proteins, such as E-cadherin, and is mainly mediated by the action of cellular fusogens called syncytins [18, 29, 30].

In parallel, based on the recent observation that human naive PSCs retain the capacity to differentiate towards trophectoderm [31, 32] and trophoblast stem cells (TSCs) [33–36], we differentiated IPSC-derived TSCs into STB [37]. Immunofluorescence analysis of TFEB subcellular localization in BeWo cells revealed that FRSK treatment significantly promotes TFEB nuclear translocation (Fig. 1D). Consistently, differentiation of BeWo cells is associated with TFEB dephosphorylation, as shown by the TFEB molecular weight shift observed in FRSK-treated cells (Fig. 1E). Also, induction of STB differentiation of BeWo cells resulted in increased expression of human chorion gonadotropin β subunit (CGB) as shown by immunofluorescence analysis via High-Content Imaging (Fig. 1F). Consistently with the results obtained in BeWo cells, we observed that STB differentiation of human TSCs is characterized by an increase in TFEB nuclear localization (Fig. 1G), further confirmed by its molecular weight shift, as measured by western blot analysis (Fig. 1H). Also, in line with the acquisition of endocrine competencies, human STB produces significantly higher levels of CGB compared to undifferentiated hTSCs (Fig. 1I).

### TFEB displays a placenta-specific genomic occupancy

**Fig. 2:**
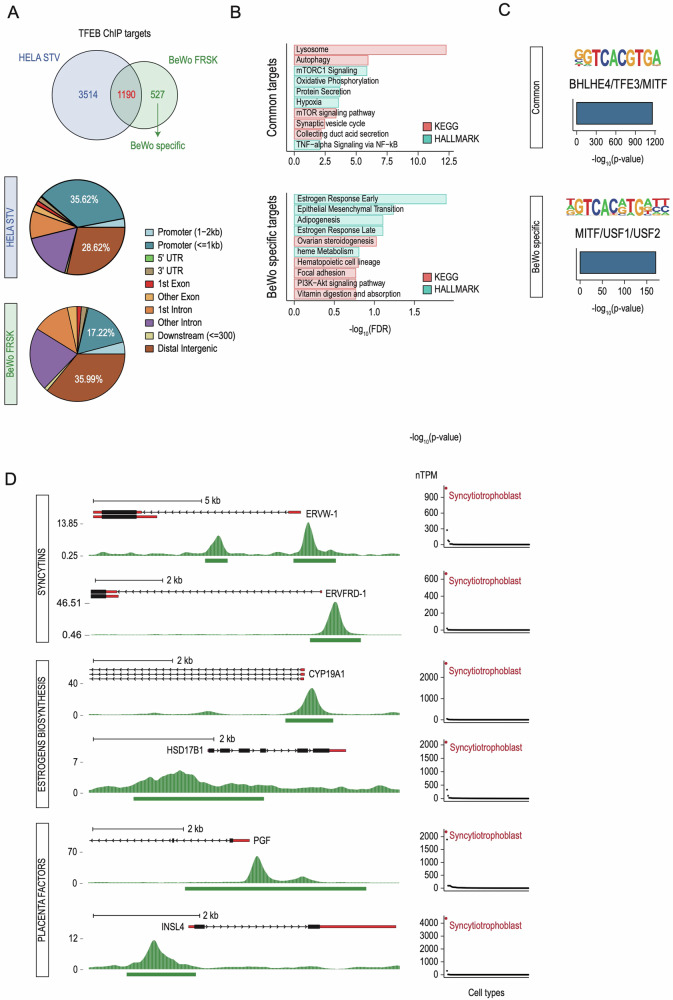
TFEB targets STB-specific genes. **A** (Upper) Venn diagram showing the number of TFEB targets (ChIP peaks at <2 kb from the TSS) in HeLa undergoing starvation (HELA STV) and BeWo cells treated with Forskolin (BeWo FRSK). Common targets (1190) are shown in red and BeWo-specific targets (527) in green. (Lower) Pie charts displaying TFEB binding distribution across the genome in HeLa and BeWo cells at the level of indicated cis-regulatory regions. The distribution of TFEB binding to different regions of target genes, which include promoters (< of 1Kb from the transcriptional start site) and distal intergenic regions, are indicated in the pie charts. **B** Bar plots of representative term enrichment analysis results using Curated Pathways (KEGG and MSigDB Hallmark collection) of TFEB target genes commonly shared between HeLa and BeWo cells (upper - Common targets) and TFEB BeWo-specific targets (lower - BeWo specific targets). Enriched terms are ranked by −log_10_ FDR (x-axis). **C** De-novo motif discovery analysis across the genomic regions displayed in **A**. For “Common” and “BeWo-specific” categories, the human-specific enriched motif is reported as a motif graph, the best match according to the Homer database, a bar plot displaying statistical significance and the percentage of targets across the genome. **D** (Left) Representative genome browser snapshots of selected promoters bound by TFEB upon Forskolin treatment in BeWo cells. Both reads distributions as density plots and peak intervals are displayed. Displayed genes are grouped into categories, indicated on the left (“Syncytins” - ERVW-1 and ERVFRD-1; “Estrogen Biosynthesis” - CYP19A1 and HSD17B1; “Placenta Factors” - INSL4 and PGF). (Right) Dot plots indicating the expression levels (nTPM) of indicated TFEB targets across different cell types according to the Human Protein Atlas single-cell datasets. Syncytiotrophoblast is indicated in red.

Having demonstrated that STB formation is characterized by increased TFEB nuclear localization, we performed chromatin immunoprecipitation sequencing (ChIP-seq) to evaluate the genomic occupancy of endogenous TFEB. To investigate whether TFEB binds to the promoters of STB-specific genes, we compared TFEB binding profiles in FRSK-treated BeWo cells versus HeLa cells undergoing starvation, chosen as the gold standard for identifying TFEB targets. Such comparative analysis highlighted that while TFEB binds 1190 genes in both cell lines (“Common targets”), a variable proportion of genes were specifically bound by TFEB in HeLa or BeWo, with 527 gene promoters uniquely bound by TFEB upon STB induction (“BeWo-specific targets”) (Fig. 2A, upper panel and Fig. S2A, B). Also, analysis of TFEB binding distribution across the genome revealed that while TFEB tends to bind more to the promoter regions of target genes in HeLa (35,62% in HeLa versus 17,22% in BeWo), it has a higher propensity of binding intergenic regions in BeWo cells (28,62% in HeLa versus 35,99% in BeWo) (Fig. 2A, lower panel).

GO analysis showed that while TFEB targets shared between HeLa and BeWo (“Common targets”) fall mainly under the “Lysosome”, “Autophagy” and “mTORC1 signaling” categories, in agreement with TFEB established function [2, 38] (Fig. 2B, upper panel), “BeWo-specific” TFEB targets include genes involved in steroid hormone production (“ovarian steroidogenesis”) and response to estrogen stimulation (“estrogen response early” and “estrogen response late”), consistent with the endocrine functions of STB (Fig. 2B, lower panel). Interestingly, in silico de novo motif analysis highlighted that while TFEB targets shared between HeLa and BeWo cells contain the canonical E-box sequence (CACGTG), BeWo-specific TFEB targets display, at the 4^th^ and 5^th^ position, a variable representation of A/G/C and T/A respectively (Fig. 2C and Fig. S2C).

FRSK treatment also induced the binding of TFEB to the promoters of ERVFRD-1 (Syncytin-2) and ERVW-1 (Syncytin-1) genes, which possess renowned fusogenic properties essential for STB formation [16, 17, 29, 39, 40] (Fig. 2D, left panel). Also, in line with the GO analysis, TFEB was enriched at the promoters of genes encoding enzymes involved in the metabolism of 17β-Estradiol (E2), such as CYP19A1 and 17β-Hydroxysteroid dehydrogenase 1 (HSD17B1) (Fig. 2D, left panel). Finally, proteins with a critical role in placenta development (PGF, INSL4) [41, 42] were also identified as TFEB targets (Fig. 2D, left panel). Notably, expression analysis using a single-cell human tissue dataset [25] showed that all the indicated TFEB targets display remarkably higher levels in STB compared with other tissues (Fig. 2D, right panel). Together, these results support the hypothesis that TFEB is involved in the formation of STB via transcriptional control of the expression of a gene network crucial for STB formation and function.

### Lack of TFEB impairs the formation of STB

**Fig. 3:**
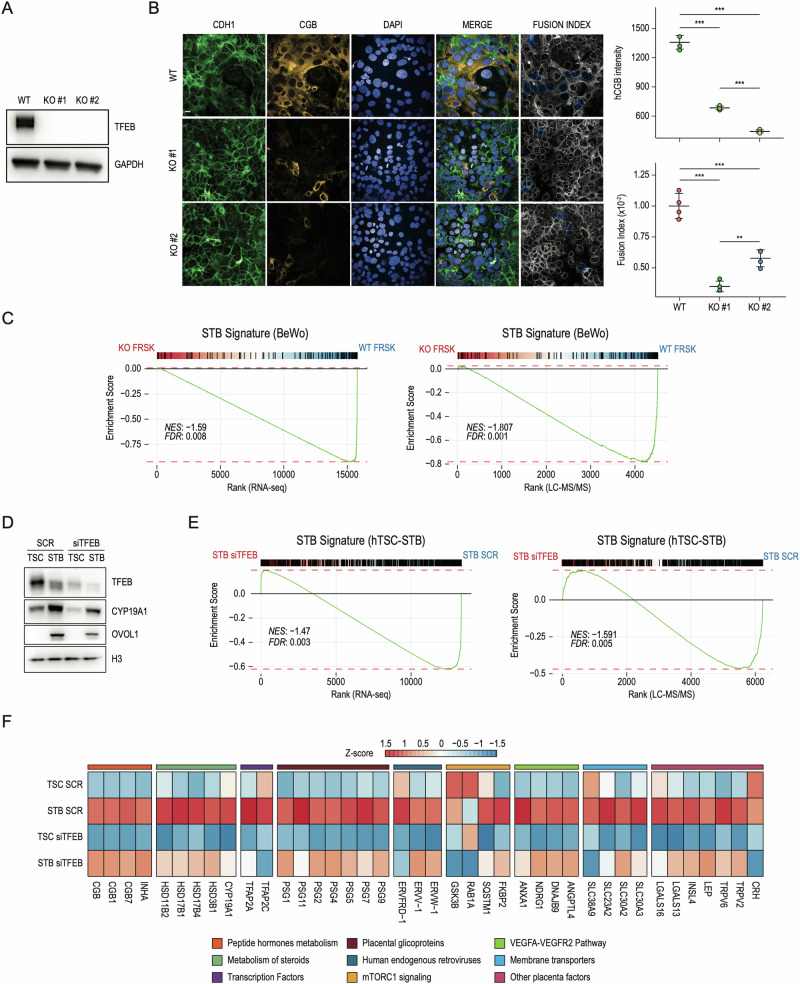
Lack of TFEB impairs STB formation. **A** Representative image of immunoblot analysis of TFEB expression in wild-type (WT) and CRISPR-Cas9 TFEB knock-out (KO#1 and KO#2) cells. GAPDH was used as a loading control. **B (**Left) Representative immunofluorescence images of wild-type (WT) cells and TFEB knock-out (KO#1 and KO#2) cells upon Forskolin (FRSK) treatment immunostained for E-Cadherin (CDH1) and human chorion gonadotropin β subunit (CGB). Nuclei are counterstained blue using DAPI. Lack of CDH1 staining combined with DAPI fluorescence was used to denote clusters of fused cells (Fusion Index, see “Methods”). Scale bar 20 µm. (*Right*) High-content imaging-based quantification of CGB intensity and proportion of fused cells upon indicated treatments (*n* = 4) shown as dot plots. Statistical analysis was performed by One-way analysis of variance (ANOVA) followed by Tukey’s multiple comparisons test (*p ≤ 0.05; **p ≤ 0.01; ***p ≤ 0.001). **C** Gene-set enrichment Analysis was performed on genes ranked by their fold-change and significance (see Methods) in FRSK-treated BeWo cells upon TFEB KO against WT cells in both RNAseq (left) and LC–MS/MS (right). Upregulated genes from panel in Fig. S1A (STB signature - BeWo) were used as geneset. Normalized Enrichment Score (NES) and False Discovery Rate (FDR) are reported. **D** Representative image of immunoblot analysis of TFEB, CYP19A1 and OVOL1 levels in human naive IPSC-derived trophoblast stem cells (TSC) and differentiated syncytiotrophoblast (STB) treated with siRNA targeting TFEB (siTFEB) and scramble sequences (SCR). H3 was used as a loading control. **E** Gene-set enrichment Analysis was performed on genes ranked by their fold-change and significance (see Methods) in syncytiotrophoblast (STB) treated with siRNA targeting TFEB (siTFEB) against human IPSC-derived trophoblast stem cells (TSC) in both RNAseq (left) and LC–MS/MS (right). Upregulated genes from panel in Fig. S1D (STB signature - TSC-STB) were used as geneset. Normalized Enrichment Score (NES) and False Discovery Rate (FDR) are reported. **F:** Heatmap of Z-scored log_2-_normalized expression values of selected differentially expressed genes in human naive IPSC-derived trophoblast stem cells (TSC) and differentiated syncytiotrophoblast (STB) treated with siRNA targeting TFEB (siTFEB) and scramble sequences (SCR). Genes displayed were grouped into categories indicated on the bottom.

To evaluate the impact of TFEB depletion on STB formation, we generated two distinct clones of BeWo cells depleted for TFEB (KO#1 and KO#2) through CRISPR-Cas9 technology (Fig. 3A) and treated them with FRSK. High-content imaging analysis revealed that CGB expression was dramatically reduced in FRSK-treated TFEB KO cells (Fig. 3B). Also, evaluation of E-cadherin (CDH1) distribution as cellular borders revealed an impairment of the formation of multinucleated syncytium, as shown by the quantification of the fusion index score (Fig. 3B). To identify the molecular mechanisms underlying the observed phenotype, we performed RNAseq and LC–MS/MS of wild-type and TFEB KO BeWo cells in DMSO and FRSK conditions. By integrating transcriptomic and proteomic data, we defined the gene signature associated with in vitro STB formation, hereafter referred to as “STB signature (BeWo)” (Fig. S1A), which revealed enrichment of pathways guiding placenta development and acquisition of syncytiotrophoblast functional properties, including “Epithelial-Mesenchymal Transition”, “Hypoxia”, “mTORC1 signaling” and “Ovarian steroidogenesis” (Fig. S1B). Notably, gene set enrichment analysis (GSEA) revealed that in the absence of TFEB, the “STB signature” was significantly downregulated at both RNA (Fig. 3C, left panel) and protein levels (Fig. 3C, right panel). Similarly to CRISPR-mediated TFEB KO, siRNA-mediated interference of TFEB expression during FRSK treatment determined a significant downregulation of STB signature (Fig. S1C).

To further strengthen the evidence of TFEB’s involvement in STB formation, we downregulated TFEB expression using siRNAs in IPSC-derived TSCs and induced them to differentiate into STB. Immunoblot analysis showed that, upon TFEB reduction, the expression levels of CYP19A1 and OVOL1, known STB markers [43], decreased (Fig. 3D). Notably, transcriptomic and proteomic analyses of TSCs and STB transfected with siTFEB revealed a significant impairment of the STB signature established from the differentiation of IPSC-derived TSCs (Fig. 3E and Fig. S1D, E - “STB signature (TSC-STB)”). Importantly, upon TFEB downregulation, the expression of numerous crucial STB genes was remarkably downregulated. These genes include: (i) proteins involved in hormone metabolism (CGB, CGB1, CGB7 and INHA); (ii) enzymes responsible of steroids metabolism (CYP19A1 [23], HSD3B1, HSD17B4, HSD17B1, HSD11B2 [44]); (iii) TFs with a role in early trophoblast progenitor specification (TFAP2A and TFAP2C) [45]; (iv) placental glicoproteins (PSG1, PSG11, PSG2, PSG4, PSG5, PSG7, PSG9); (v) fusogenic proteins (ERVW-Q, ERVV-Q and ERVFRD-1); (vi) components of mTORC1 pathway (GSK3B, RAB1A, SQSTM1, FKBP2); (vii) factors of the VEGFA-VEGFR2 pathway (ANGPTL4, DNAJB9, NDRG1, ANXA1); (viii) membrane transporters (SLC30A2, SLC30A3, SLC23A2, SLC38A9) and ix) placenta specific factors (CRH, TRPV2, TRPV6, LEP, INSL4, LGALS13, LGALS16) (Fig. 3F).

Importantly, although we employed two distinct in vitro cellular models to investigate TFEB’s role in STB, the comparison of the corresponding gene signatures highlighted 65 commonly upregulated genes, which include crucial STB markers such as CGA, CYP11A1, CYP19A1, ENG, and SYDE1 (Fig. S1F), further reinforcing the role of TFEB in STB formation.

Interestingly, to further prove the key role of TFEB in STB, we treated BeWo cells with Torin-1, a potent mTORC1 inhibitor known to promote TFEB nuclear translocation [6] and observed increased CGB expression, in agreement with the promotion of differentiation (Fig. S3A). Conversely, Rapamycin, which inhibits mTORC1 without inducing TFEB nuclear translocation [6], did not affect CGB expression (Fig. S3A). Accordingly, qPCR analysis revealed that while Torin-1 increases the expression of selected STB markers similarly to FRSK, Rapamycin did not (Fig. S3B). Also, Torin-1 treatment of TFEB KO BeWo cells could not promote STB formation, as shown by the abrogation of CGB induction (Fig. S3C), indicating that mTORC1 inhibition promotes STB formation through the induction of TFEB nuclear translocation. Collectively, these data strongly suggest that TFEB is an essential regulator of STB formation.

### TFEB promotes STB endocrine functions

**Fig. 4:**
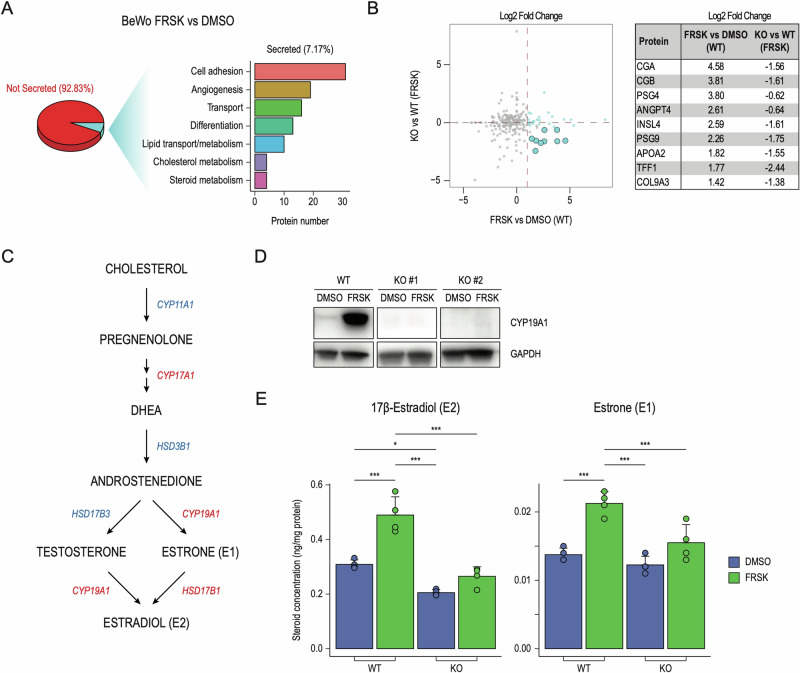
TFEB drives STB endocrine functions. **A** Analysis of secreted proteins through LC–MS/MS in wild-type (WT) and TFEB knock-out (KO) cells. (Left) Pie chart showing the percentage of detected proteins classified into “not secreted” (92.83%) and “secreted” (7.17%) according to Human Protein Atlas. (*Right*) Bar plot showing the number of secreted proteins (x-axis) belonging to the indicated Biological Process categories (y-axis). **B** (Left) Volcano plot showing secreted proteins displayed as Log_2_ Fold Change in wild-type (WT) cells upon Forskolin (FRSK) treatment compared to DMSO conditions - FRSK vs DMSO (WT) on the x-axis - and in TFEB knock-out (KO) cells treated with FRSK with respect to WT in the same conditions - KO vs WT (FRSK) on the y-axis. Proteins upregulated upon FRSK treatment in WT cells and downregulated in KO versus WT cells in FRSK conditions are represented as light blue dots. (Right) Table displaying selected proteins and their corresponding Log_2_ Fold Change in indicated conditions. **C** Schematic representation of the estrogen biosynthetic pathways from cholesterol to 17β-Estradiol (E2). Major enzymes synthesizing indicated steroid intermediates are displayed in red (directly bound by TFEB from our ChiPseq analysis) and in blue (not directly bound by TFEB but affected by its downregulation). **D** Representative image of immunoblot analysis of CYP19A1 levels in cells wild-type (WT) and TFEB knock-out cells (KO#1 and #2) in DMSO e Forskolin (FRSK) conditions. GAPDH was used as a loading control. **E** Bar chart graph representations of the quantitative determination of 17β-Estradiol (E2) and Estrone (E1) in wild-type (WT) and TFEB knock-out (KO) cells in DMSO and Forskolin (FRSK) conditions detected by UHPLC–MS/MS-based targeted steroidomics. Steroid concentration values are expressed as ng/mg of protein. Statistical analysis was performed by One-way analysis of variance (ANOVA) followed by Tukey’s multiple comparisons test (*p ≤ 0.05; **p ≤ 0.01; ***p ≤ 0.001).

STB is the site with the highest metabolic and endocrine activity in the placenta. It secretes proteins and hormones into the maternal systemic circulation that are essential for pregnancy establishment and maintenance, exerting autocrine and paracrine effects that regulate placental and embryo implantation [23]. Our data showed that TFEB controls the expression of several hormones and enzymes involved in hormone metabolism (Fig. 2D). We analyzed the secretome of FRSK-treated BeWo cells and observed the presence of secreted proteins involved in diverse biological functions, spanning from cell adhesion, angiogenesis, transport and differentiation (Fig. 4A). In line with the acquisition of endocrine activity, STB secretes several hormonal peptides (CGA, CGB, and INSL4), placenta-specific glycoproteins (PSG4, PSG9), and proteins with essential roles in vascular development and angiogenesis (ANGPT4). Notably, FRSK-treated TFEB KO cells displayed an impairment in the secretion of these proteins, further reinforcing the critical role of TFEB in regulating STB function (Fig. 4B).

**Fig. 5:**
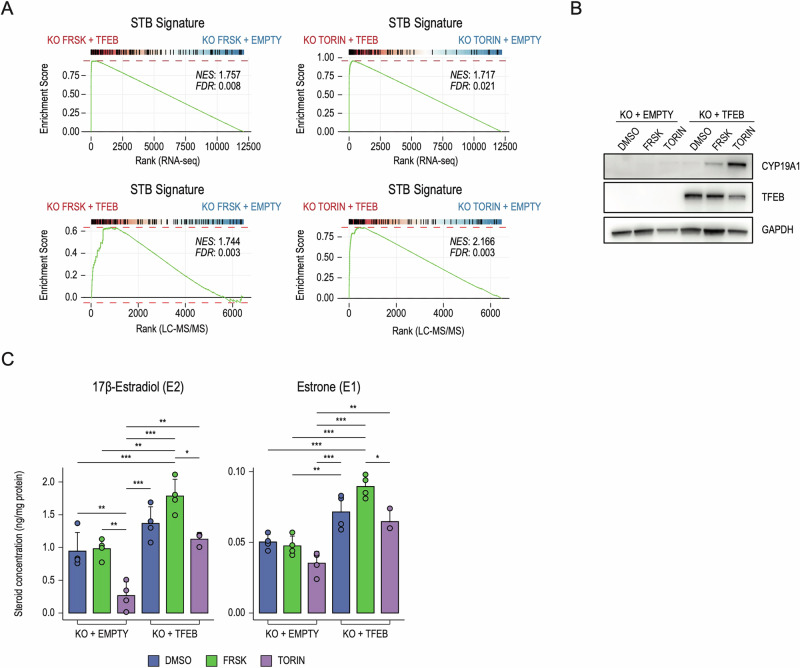
TFEB overexpression restores the STB phenotype in TFEB KO cells. **A** Gene-set Enrichment Analysis was performed on genes ranked by their fold-change and significance (see “Methods”) in TFEB knock-out cells transduced with a TFEB overexpressing vector (KO + TFEB) against an empty control vector (KO + EMPTY), upon treatment with Forskolin (FRSK - left) or Torin-1 (TORIN - right) in RNAseq (upper panels) and LC–MS/MS (lower panels). Upregulated genes from panel S1A (STB signature - BeWo) were used as geneset. Normalized Enrichment Score (NES) and False Discovery Rate (FDR) are reported. **B** Representative image of immunoblot analysis of CYP19A1 and TFEB levels in TFEB knock-out cells transduced with an empty control vector (KO + EMPTY) or TFEB overexpressing vector (KO + TFEB) upon treatment with DMSO, Forskolin (FRSK) and Torin-1 (TORIN). GAPDH was used as a loading control. **C** Bar chart graph representations of the quantitative determination of 17β-Estradiol (E2) and Estrone (E1) in TFEB knock-out cells transduced with an empty control vector (KO + EMPTY) or TFEB overexpressing vector (KO + TFEB) upon treatment with DMSO, Forskolin (FRSK) and Torin-1 detected by UHPLC–MS/MS-based targeted steroidomics. Steroid concentration values are expressed as ng/mg of protein. Statistical analysis was performed by One-way analysis of variance (ANOVA) followed by Tukey’s multiple comparisons test (*p ≤ 0.05; **p ≤ 0.01; ***p ≤ 0.001).

Besides peptide hormones, STB also secretes steroid hormones, like estradiol E2, which regulates vascular development and blood-flow dynamics of both uterus and placenta throughout gestation by stimulating uterine angiogenesis [46–48]. Placental estrogen biosynthesis consists of a series of enzymatic reactions that lead to the synthesis of E2 from both testosterone and estrone (E1) through CYP19A1 and HSD17B1, respectively [24] (Fig. 4C). We discovered that TFEB directly regulates the expression of CYP19A1, also known as aromatase, which is the rate-limiting enzyme for the production of E2 [49] (Fig. 2D). Furthermore, TFEB KO in FRSK-treated BeWo cells entirely abrogated CYP19A1 expression, as measured by immunoblot (Fig. 4D), RNAseq and LC–MS/MS analysis (Fig. 3C). Accordingly, while FRSK promoted a significant increase in E2 synthesis in wild-type cells, TFEB depletion abrogates E2 induction (Fig. 4E, left panel). In line with CYP19A1 function, E1 synthesis was also impaired in the absence of TFEB (Fig. 4E, right panel). Also, TFEB depletion increased cortisol and decreased cortisone levels due to reduced HSD11B2 expression (Fig. S4A). Finally, overexpression of TFEB in TFEB KO BeWo cells followed by treatment with FRSK or Torin-1 (Fig. S4B) induced a significant rescue of the STB signature at RNA and protein levels (Fig. 5A). Accordingly, CYP19A1 expression was restored in TFEB-overexpressing cells (Fig. 5B) with consequent reactivation of E1 and E2 synthesis (Fig. 5C). Also, TFEB overexpression determined a decrease in cortisol and an increase in cortisone levels, suggesting a restoration of HSD11B2 activity (Fig. S4C). Collectively, these results reveal a central role for TFEB in controlling the placental endocrine properties.

## Discussion

The fusion of biological membranes is of fundamental relevance in diverse physiological processes, including organelle biogenesis and vesicle trafficking, as well as developmental phenomena, such as placental formation [30]. Indeed, most human trophoblast transport capabilities and endocrine activities are associated with the syncytial phase of trophoblast differentiation. Indeed, changes in the placental transport capacity constitute a direct cause of altered fetal growth [50]. The critical involvement of TFEB in placental development was first proposed by Steingrimsson et al., who highlighted that homozygous TFEB KO mice displayed defects in the ability of the embryonic vasculature to invade the placenta, suggesting that impairment of oxygen and proper nutrient exchange could be at the base of the observed lethality [9]. The involvement of TFEB in controlling vascular development was also demonstrated in endothelial cells by different groups [10, 51] and, in line with these observations, we also detected deregulation of the VEGFA-VEGFR pathway in TFEB KO placental cells. However, whether or not TFEB had a specific role in placental development has remained unclear for more than 20 years after the intriguing original observation of placental defects in TFEB KO mice [9].

Our in vivo analyses revealed that the absence of TFEB in homozygous TFEB KO mice determines reduced SynTII formation, prompting us to investigate the molecular mechanism underlying TFEB’s role in the syncytiotrophoblast by employing human cellular in vitro models of syncytialization. Here, we demonstrated that FRSK-induced differentiation of BeWo cells and differentiation of IPSC-derived human trophoblast stem cells into STB promotes TFEB nuclear translocation. Interestingly, ChIPseq analysis revealed that TFEB binds to the promoters of placental-specific genes that play prominent roles in STB identity. Thus, we observed that TFEB-specific placenta targets contain, in their promoter, an E-box sequence that slightly differs from the canonical one, suggesting that TFEB might control tissue-specific gene programs depending on the DNA sequence motifs. Integrating multi-omic approaches with cell-based functional and phenotypic assays enabled us to establish an unprecedented role for TFEB as an essential determinant of the fusion, identity and function of the syncytiotrophoblast. Interestingly, in line with our observation, a very recent work identified, through a CRISPR screening in human trophoblast stem cells, TFEB as one of the transcription factors relevant for syncytiotrophoblast formation [52].

Following TFEB depletion, cells stimulated with FRSK and IPSC-derived human trophoblast stem cells differentiated into STB fail to induce an STB-specific signature, as measured by RNAseq and proteomics analysis. Indeed, the expression of many STB-specific genes were significantly impaired following the reduction of TFEB. These include the downregulation of ERVW-1 and ERVFRD-1 [17], identified in this work as TFEB targets, with consequent reduction of cell fusogenic capacities.

Accordingly, evaluation of E-Cadherin distribution at cellular borders confirmed an impairment of the formation of multinucleated syncytium, suggesting that the epithelial-mesenchymal transition process is affected by TFEB depletion. This is in line with the observation that TFEB regulates EMT in mouse epicardial cells [53].

Previous studies have established the role of TFEB in the regulation of lipid metabolism [54]. Notably, in vascular endothelial cells, TFEB exerts metabolic control over endogenous cholesterol synthesis and the pro-endocytic function of CAV-1, impacting endothelial cells adhesion to the extracellular matrix, which is crucial for angiogenesis [55]. This observation might also provide a valuable explanation to the vascular defects observed in the TFEB-KO placenta, as reported by Steingrímsson and colleagues which warrants further investigation.

In the placenta, the glucocorticoid-inactivating enzyme HSD11B2 converts biologically active cortisol into inactive cortisone, acting as the placental glucocorticoid barrier to protect the fetus from the adverse effects of excessive maternal cortisol [56] and impairment of this placental glucocorticoid barrier is associated with fetal IUGR and development of chronic diseases in later life [57, 58]. We showed that in TFEB-depleted cells, the expression of HSD11B2 is considerably downregulated, with a consequent increase in cortisol levels. TFEB overexpression in TFEB KO cells reduced cortisol levels and increased cortisone production, suggesting that HSD11B2 activity is restored and demonstrating a relevant role of TFEB in balancing the placental glucocorticoid barrier.

We also discovered that TFEB regulates the expression of CYP19A1, also known as aromatase, which catalyzes the final and critical step of the 17β-Estradiol (E2) synthesis cascade by mediating the conversion of C19 steroids to estrogens [49]. In the absence of TFEB, CYP19A1 expression is entirely abolished, with a consequent reduction of E1 and E2 production. Notably, overexpression of TFEB in TFEB KO cells restored STB identity, with the consequent rescue of CYP19A1 expression and estrogens production. These data establish an entirely novel function of TFEB in controlling the placenta steroidogenic program, which is crucial for the promotion of angiogenesis, trophoblast invasion, and synthesis of nitric oxide and placental-specific factors [59–62]. This discovery might have relevant implications in pregnancy-associated diseases, including pre-eclampsia - one of the primary causes of maternal and fetal mortality and morbidity worldwide -, where defects in placentation and/or disturbance of estrogens metabolism play a fundamental role [63, 64]. Further studies will be required to dissect the role TFEB-CYP19A1 transcriptional axis in pregnancy-associated diseases and to determine whether TFEB could act as a general transcriptional regulator of endocrine properties in other specialized steroidogenic organs, including ovaries, testis, and adipose tissue. Collectively, our findings elect TFEB as a crucial determinant of placental development via the regulation of critical steps of STB differentiation.

## Methods

### Mouse strains and immunofluorescence staining of E10.5 placentas

All procedures on mice were approved by the Italian Ministry of Health. Mice were housed at the TIGEM animal house under SPF certification. For the generation of TFEB KO mice, B6.C-Tg(CMV-cre)1Cgn/J mice from Jackson laboratories (Stock No: 006054) were crossed with TFEB conditional KO mice [6].

Heterozygotes derived from these crosses were crossed with each other to obtain the full KO. The genotyping of heterozygous and KO mice was performed by PCR.

Placentas were dissected from pregnant females at E10.5, washed briefly in ice-cold PBS and fixed in 4% PFA overnight. The next day, placentas were embedded in paraffin and sectioned (15 μm). Slides were deparaffinized in Sub-X and ethanol. Antigen retrieval was promoted by heating the slides in a citrate buffer, pH 6,0 at 90 C for 10 min. After permeabilization in PBS −0,3% Triton X-100 for 5 min, non-specific sites were blocked in 10% horse serum in PBS-0,1% Triton X-100 for 1 h at room temperature and incubated overnight at 4 °C with primary antibodies (rabbit anti-Mct4, Millipore, AB3314P) diluted with 5% horse serum in PBS-0,1% Triton X-100. The next day, sections were stained with the appropriate secondary antibodies for 1 h at room temperature, washed in PBS. Nuclei were visualized by DAPI staining. At least three specimens per genotype were tested and representative fields are presented. Mct4 “Mean Instensity” and “Area” signal quantification was performed using ImageJ software on three distinct sequential sections per animal.

### Cell culture

BeWo cells were obtained from ATCC and cultured in F-12K medium (Kaighn’s Modification of Ham’s F-12 Medium) (ATCC, # 30-2004,) supplemented with 10% FBS (Euroclone, # ECS0186L), penicillin (100 IU/ml) and streptomycin (100 μg/ml) (Euroclone, # ECB3001D,). To stimulate BeWo trophoblast differentiation, cells were treated with 100 μM of Forskolin (Sigma-Aldrich, #F6886), which is commonly used to stimulate syncytialization in these cells. All cells were maintained at 37 °C, 5% CO_2_.

hTSCs were cultured as described by Okae et al., 2018 [65]. Briefly, a 6-well plate was coated with 5 μg/mL Collagen IV (Corning, # 354233) at 37 °C overnight and cells were cultured in in TS medium (DMEM/ F12 supplemented with 0.1 mM 2-mercaptoethanol, 0.2% FBS, 0.5% Penicillin–Streptomycin, 0.3%, BSA [Gibco 15260-037], 1% ITS-X [Gibco, 51500], 1.5 μg/mL l-ascorbic acid [Sigma, # A4544], 50 ng/ml EGF [Peprotech, # AF-100-15], 2 μM CHIR99021 [Axon Medchem, # 1386 and 1408], 0.5 μM A83-01 [Axon Medchem, #1421], 1 μM SB431542 [Axon Medchem, # 1661], 0.8 mM VPA [HDACi, Sigma, #P4543], and 5 μM Y-27632 (Axon Medchem). Media were changed every 2 days, and cells were passed using TrypLE Express (Gibco, # 12604013) every 3–4 days at a ratio of 1:8. Differentiation of TSCs into syncytiotrophoblast (STB) was performed according to Zorzan et al., 2023. Briefly, 6-well plates were coated with 2.5 ug/mL Collagen IV overnight. 2 × 10^5^ TSCs were seeded per well in 2 mL STB medium (DMEM/F12 supplemented with 0.1 mM b-mercaptoethanol, 0.5% penicillin–streptomycin, 0.3% BSA, 1% ITS- X, 2.5 μM Y-27632, 2 μM Forskolin and 4% KSR).

### Immunofluorescence and high-content imaging analysis

2 ×10^5^ cells were plated on 96-well plates (PerkinElmer, ViewPlate, #6055302) and subjected to the indicated treatments. Then, cells were washed twice with PBS Dulbecco’s w/o Calcium w/o Magnesium (Euroclone, # ECB4004) and fixed with 4% PFA at room temperature for 10 min. Preliminary permeabilization with 0.1% PBS Triton solution was performed and then blocked with PBS 3% BSA solution for 1 h at room temperature. Overnight incubation was carried out with the following primary antibodies: E-Cadherin (Cell Signaling, #24E10), hCGB (Abcam [5H4-E2], #AB9582) and TFEB (Cell Signaling, #4240s). Secondary antibodies Alexa Fluor 488 (donkey anti-rabbit) and 568 (donkey anti-mouse) were used for protein staining, and Hoechst solution for nuclei visualization. Images were acquired with the OPERA Phenix High Content Screening (PerkinElmer). Images were analyzed with Columbus image analysis software. To evaluate TFEB nuclear translocation, the intensity of TFEB in both the nucleus and cytoplasm was measured and expressed as a ratio. For the quantification of CGB expression, the intensity of CGB signal was measured and normalized to the number of nuclei. To measure the fusion capability (Fusion Index) of BeWo cells upon the indicated treatments, morphological parameters of nuclei were utilized to discriminate fusing nuclei and normalize them with the total number of cells.

### Western blot

Total cell extracts were obtained using SDS Lysis Buffer (10 mM TRIS-HCl pH8, 0.2% SDS) with the addition of protease inhibitors (Sigma Aldrich, P8340) and sonicated briefly with Bioruptor. Protein concentration was measured by BCA assay (Pierce™ BCA Protein Assay Kit). Then, 15 μg of lysate was used for Western Blotting.

The proteins are resolved on precast gradient gel 4-12% (Invitrogen, NuPAGE™ 4 to 12%, Bis-Tris, 1.0–1.5 mm, Mini Protein Gels, cat# NP0323PK2) and then transferred to PVDF membrane.

PVDF membranes were blocked with 5% milk in TBST 0.1% for 1 hr at RT. Primary antibodies (in TBST 0.1% BSA 3%) were incubated overnight at 4 °C (TFEB, Cell Signaling, # 4240 s; E-Cadherin, Cell Signaling, # 24E10; GAPDH, Santacruz, # sc32233; CYP19A1, Cell Signaling, # 14528; OVOL1, Aviva Systems Biology, #ARP38500T100). Membranes were incubated for 1 h at RT with HRP-conjugated secondary antibodies (TBST 0.1%, milk 5%). Each step was interspersed with the washing procedure in TBST 0.1%. Images were digitally acquired using an ImageQuant LAS4000 (GE Healthcare).

For the nucleus/cytoplasm fractionation, two buffer solutions were used (A: Tris HCl pH 7.4 20 mM, EDTA 0.1 mM, MgCl2 2 mM, NP40 1%; B: HEPES pH 7.4 20 mM, NaCl 400 mM, EDTA 1 mM, DTT 0,5 mM; both of them supplemented with protease and phosphatase inhibitors). After fractionation with Buffer A, nuclei were separated, resuspended with Buffer B, and lysed with thermal shock. Nuclei and Cytoplasm fractions were quantified and processed as mentioned above.

### RNA extraction, cDNA synthesis, and RT-PCR

Total RNA was isolated using the RNeasy Plus mini kit (Qiagen) according to the manufacturer’s instructions. cDNA was synthesized using the QuantiTect Reverse Transcription kit (Qiagen). qPCR was performed with the LightCycler 480 SYBR Green I mix (Roche) using the LightCycler 480 II detection system (Roche). QuantiTect Primer Assays from Qiagen were used to quantify genes of interest. HPRT1 (Hs_HPRT1_1_SG QuantiTect Primer Assay, Qiagen, QT00059066) and RPL22 (Hs_RPL22_1_SG QuantiTect Primer Assay, Qiagen, QT00079982) were used as an endogenous housekeeping control, and results were displayed as fold change compared to control samples. The following probes were used to quantify: TFEB (Hs_TFEB_1_SG QuantiTect Primer Assay, Qiagen, QT00069951)

### siRNA transfection

Transfection of siRNAs was performed in suspension using Lipofectamine RNAiMAX Transfection Reagent (cat. no. 13778, Invitrogen). Human TFEB (Dharmacon, L-009798-00-0010) and non-targeting siRNA pool (Dharmacon, D-001810-10-20) were used at the final concentration of 20 nM. Knock-down efficiency was assessed after 72 h through immunoblot and qPCR analysis.

### Generation of TFEB BeWo knock-out cells

BeWo cells knock-out for TFEB were generated using the CRISPR/Cas9 system. The gRNA sequence (CCCAGAAGCGAGAGCTCAC) with a low off-target score has been selected using the http://crispor.tefor.net/crispor.py tool. An “ALL in One” vector expressing Cas9, the specific gRNA, and GFP was obtained from SIGMA and transfected in BEWO cells using Lipofectamine Stem Transfection Reagent (Invitrogen, # STEM00008). After 48 h of transfection, GFP-positive cells were FACS-sorted into 96-well plates to obtain single-cell derived colonies, and clones carrying the INDEL mutations were identified by DNA Sanger sequencing. Primer sequences for the screening of the clones are the following: *TFEBEX4u* TTGCCTCACATCTCTGCTCA; *TFEBEX4l* AGGTTCCATGCCTTAACCCAG.

### Viral transduction and lentiviral infection

cDNA sequence for TFEB overexpression was cloned into pLVX-EF1a-PURO plasmid (Clontech) lentiviral backbone, using Infusion HD Cloning Kit (Clontech). An empty pLVX-EF1a-PURO plasmid was used as a control. Lenti-X 293T cell line (Takara) was used to produce lentiviral particles using standard procedures [66]. For viral transduction, cells were seeded at a density of 3 × 10^5^ cells per well in a 6-well plate, and viral particles were added at a multiplicity of infection (MOI) of 2 to 5 in a final volume of 1 mL in the presence of polybrene at 8 μg/mL final concentration. Media was changed 24 h post infection, and cells were selected using puromycin at a final concentration of 1 μg/mL starting from 48 h post infection.

### Chromatin immunoprecipitation library preparation and sequencing

2 ×10^7^ cells were fixed with 1% formaldehyde for 15 min at room temperature. Samples for ChIP-seq were prepped as previously described [66]. Libraries were prepared from 10 ng of DNA using the NEBNext UltraTM II DNA Library Prep Kit for Illumina (New England Biolabs). The quality of libraries was assessed using Bioanalyzer DNA Analysis (Agilent Technologies) and quantified using the Qubit 4 Fluorometer (Thermo Fisher Scientific). Libraries were sequenced on a NovaSeq 6000 sequencing system using a paired-end (PE) 100 cycles flow cell (Illumina Inc.).

### ChIP-sequencing bioinformatic analyses

Paired sequencing reads were aligned on human GRCh38reference genome using BWA [67] and filtered with samtools [68] to remove unmapped read pairs, not primary alignment, reads failing platform quality, with mapping quality score below 30, and duplicate reads were then removed using Picard MarkDuplicates (“Picard Toolkit.” 2019. Broad Institute, GitHub Repository. https://broadinstitute.github.io/picard/) (v.2.18.27). Two biological replicates were generated and a third one was generated by a pseudoreplicate approach. Peaks were called with MACS2 [68] with *p* < 0.1 on both samples and pseudoreplicates and filtered after Irreproducible Discovery Rate analysis with a threshold of 0.05. The coverage signal profile was generated with deep tools [69] using CPM normalization.

### RNA-sequencing library preparation and sequencing

Total RNA was quantified using the Qubit 2.0 fluorimetric Assay (Thermo Fisher Scientific). Libraries were prepared from 250 ng of total RNA using the 3’DGE mRNA-seq sequencing service (TIGEM NGS Core & NEGEDIA Srl), which included library preparation, quality assessment, and sequencing on a NovaSeq 6000 sequencing system using a single-end, 100-cycle strategy (Illumina Inc.).

### RNA-sequencing data pre-processing and analysis

The raw data were analyzed by NEGEDIA Srl proprietary 3′DGE mRNA-seq pipeline (v2.0), which involves a cleaning step by quality filtering and trimming, alignment to the reference genome and counting by gene. Data were normalized via the *cpm* function from the edgeR package (v. 3.34.1). Differential expression analyses were performed using edgeR (v. 3.34.1) [70] on genes having more than 1 CPM in more than the minimum number of samples belonging to one condition minus 1 and less than 20% of multi-mapping reads, simultaneously. In general, if not otherwise indicated, genes were considered differentially expressed when displaying FDR < 0.05 and log_2_ fold change >0 (upregulated) or <0 (downregulated). Pathway enrichment analysis was conducted using the enrichR (v. 3.0) package [71–73]

### UHPLC–MS/MS-based targeted steroidomics analysis

#### Chemicals

LCMS grade water (H_2_O) and methanol (CH_3_OH) were purchased by Merck (Milan, Italy), Ammonium fluoride (NH_4_F) was purchased by VWR, 17β-Estradiol (E2), 17β-Estradiol-D_2_, Estrone (E1), Estrone-D_2_, Cortisol, Cortisol-D_4_ and Cortisone standards were purchased by Cayman Chemical Company (Michigan, USA). Unless stated otherwise other reagent were all purchased by Merck.

#### Sample preparation

For steroids extraction, the cell pellets were thawed on ice and 10 μL of a mixture of internal standards (IS) 17β-Estradiol-D_2_, Estrone-D_2_ and Cortisol-D_4_ (each IS concentration was 100 ng/mL in MeOH) was added. The samples were treated with 900 μL of ice-cold MeOH/MTBE (1:3), vortexed at room temperature for 30 s and then treated in ultrasonic bath for 10 min. The solutions were mixed in a thermomixer^®^ (Eppendorf) for 15 min at 10 °C, 900 rpm and finally centrifuged at 14 680 rpm, for 10 min at 4 °C. The supernatants were recovered and were dried under vacuum for 2 h at 35 °C (Savant SPD140DDA SpeedVac Concentrator connected to a RVT5105 Refrigerated Vapout Trap, Thermo Scientific, UK). The extracts were reconstituted in 0.100 mL of H_2_O/MeOH (1:1) and then analyzed by ultra-high performance liquid chromatography-tandem mass spectrometry (UHPLC–MS/MS) operating in multiple reaction monitoring (MRM) mode.

#### UHPLC–MS/MS setup

The analyses were performed using a Shimadzu Nexera UHPLC (Kyoto, Japan) consisting of two LC-30AD pumps, a SIL-30AC autosampler, a CTO-20AC column oven and a CBM-20A controller. The chromatographic system was coupled online to a triple quadrupole LCMS-8050 (Shimadzu, Kyoto, Japan) equipped with an Electrospray Ionization (ESI) source. A Kinetex^®^ Biphenyl Column 100 × 2.1 mm, 2.6 μm (Phenomenex^®^, Bologna Italy) was used for steroid separation. Column oven temperature was set at 45 °C; flow rate was set to 0.5 mL/min; mobile phases composition was as follows: (A) H_2_O + 0.2 mM NH_4_F, (B) MeOH and the following gradient has been employed: 0 min, 20% B, 0.01–4.00 min, 20–60% B, 4.01–6.00 min, 60-70% B, 6.0.1–6.30 min, 70–99% B, isocratic for 2 min. Returning to 20% B in 0.10 min. 2 μL of extract were injected. The ESI was operated in negative and positive mode. MRM transitions for each compound were determined: 17β-Estradiol (E2), 271.0 > 185.25 (quantifier ion) and 271.0 > 145.20 (qualifier ion); 17β-Estradiol-D_2_, 273.0 > 185.30 (quantifier ion) and 273.0 > 147.20 (qualifier ion); Estrone (E1), 269.0 > 145.25 (quantifier ion) and 269.0 > 143.25 (qualifier ion); Estrone-D_2_, 271.0 > 147.25 (quantifier ion) and 271.0 > 145.20 (qualifier ion) Cortisol, 362.9 > 121.15 (quantifier ion) and 362.9 > 327.2 (qualifier ion); Cortisol-D_4_, 367.0 > 121.15 (quantifier ion) and 367.0 > 331.15 (qualifier ion); Cortisone, 360.9 > 163.20 (quantifier ion) and 360.9 > 121.15 (qualifier ion). Interface temperature, Desolvation line temperature, Heat Block temperature were set, respectively to 300 °C, 250 °C and 400 °C. Nebulizing gas, drying (N_2_) and heating gas (air) were set, respectively, to 3, 10 and 10 L/min.

#### Steroid quantification and method validation

Stock solutions (1 mg/mL) of each authentic standard 17β-Estradiol (E2), Estrone (E1), Cortisol and Cortisone were prepared in MeOH and were further diluted in H_2_O/MeOH (1:1) to prepare a mixture of standards of suitable concentrations. IS were added at concentrations of 100 ng/mL. The calibration curve was obtained in the concentration range of 0.1–100 ng/mL (*R*^2^ = 0.999). The samples were analyzed in a random order to prevent any possible bias due to variation in mass spectrometer sensitivity. Moreover, Quality Control (QC) was randomly inserted in the batch to monitor system stability over time. For sensitivity evaluation, Limits of detection (LODs) and quantification (LOQs) were calculated by the ratio between the standard deviation (SD) and analytical curve slope multiplied by 3 and 10, respectively. Repeatability was established by triplicate injections of QC sample with the same chromatographic conditions and analyst on the same day, and within a consecutive day, and results were expressed as relative standard deviation % (RSD %) for concentration and retention time.

Recovery was assessed by spiking a known amount of IS at low, medium and high concentration range to control samples, which were subsequently extracted and analyzed as previously.

#### Statistical analysis

Statistical analysis was performed using GraphPad prism 8.0 software for Windows (GraphPad Software, San Diego, CA). One-way analysis of variance (ANOVA) followed by Tukey’s multiple comparisons test was used to evaluate the differences of mean values of the molecules among the sample groups. Analyses with *p* ≤ 0.05 were considered statistically significant.

### LC–MS/MS mass spectrometry

All the experiments have been performed in a labeling-free setting. Samples were processed using iST-KIT (Preomics). For the proteomic and secretome experiments, 4 × 10^5^ BeWo WT and BeWo-TFEB KO cells were seeded in a 6-well plate in 2 mL of medium. The day after, the medium was replaced with a fresh one containing DMSO and Forskolin (100 μM) to induce syncytiotrophoblast differentiation for 48 h. For proteomic experiments, cells were washed twice with PBS Dulbecco’s w/o Calcium w/o Magnesium (Euroclone, # ECB4004) and lysed in SDS 0.2% TRIS HCl pH8 10 mM.

For the secretome experiment, after 48 h of differentiation, the cells were washed twice with PBS Dulbecco’s w/o Calcium w/o Magnesium (Euroclone, # ECB4004), and the culture medium was replaced with Opti-MEM^TM^ (Gibco, # 31985062) containing DMSO and Forskolin (100 uM). After an additional 24 h, the medium was harvested and spun down at 600 × *g* for 5 min at +4 °C to eliminate cell debris.

Proteins were precipitated with ice-cold Acetone (10 μg for proteome and 50 μg for secretome experiments). The samples were denaturated with 6 M Guanidine Hydrochloride 50 mM Tris pH8.5 5 mM TCEP 20 mM CAA solution and boiled for 10 min at 95 °C. Proteins were digested with Lys-C/Trypsin. A procedure with stage tips (using C18 disc) was performed. Activation, washing, and elution solutions are Methanol, Buffer A (0.1% Formic Acid) and Buffer B (80% Acetonitrile, 0.1% Formic Acid).

LC–MS/MS analysis instruments consisted of a NanoLC 1200 coupled via a nano-electrospray ionization source to the quadrupole-based Q Exactive HF benchtop mass spectrometer. Peptide separation was carried out according to their hydrophobicity on a home-made chromatographic column, 75 mm ID, 8Um tip, 350 mm bed packed with Reprosil-PUR, C18-AQ, 1.9 mm particle size, 120 Angstrom pore size, using a binary buffer system consisting of solution A: 0.1% formic acid and B: 80% acetonitrile, 0.1% formic acid.

Runs of 120 min after loading were used for proteome samples (DDA), with a constant flow rate of 300 nl/min. Runs of 60 min after loading were used for proteome samples (DIA), with a constant flow rate of 300 nl/min.

Q Exactive HF settings: MS spectra were acquired using 3E6 as an AGC target, a maximal injection time of 20 ms and a 120,000 resolution at 200 m/z.

The mass spectrometer operated in a data-dependent Top20 mode with the subsequent acquisition of higher-energy collisional dissociation (HCD) fragmentation MS/MS spectra of the top 20 most intense peaks. Resolution for MS/MS spectra was set to 15,000 at 200 m/z, AGC target to 1E5, max injection time to 20 ms and the isolation window to 1.6Th. The intensity threshold was set at 2.0 E4 and Dynamic exclusion at 30 s.

### LC–MS/MS data analysis

All experiments were performed in at least three independent biological replicates. Data are presented as mean ± e.v. Statistical analysis of two experimental groups was performed using parametric two-tailed Student’s *t*-test.

For mass spectrometry: (i) DDA-acquired RAW files were processed using MaxQuant and the implemented Andromeda search engine; (ii) DIA acquired RAW files were processed using DIANN. For protein assignment, spectra were correlated with the UniProt human database (v. 2023) including a list of common contaminants. Searches were performed with tryptic specifications and default settings for mass tolerances for MS and MS/MS spectra. Carbamidomethyl at cysteine residues was set as a fixed modification, while oxidations at methionine, acetylation at the N-terminus were defined as variable modifications. The minimal peptide length was set to seven amino acids, and the false discovery rate for proteins and peptide-spectrum matches to 1%. The match-between-run feature with a time window of 1 min was used. For further analysis, the Perseus software (1.6.2.3) was used and first filtered for contaminants and reverse entries as well as proteins that were only identified by a modified peptide. The LFQ Ratios were logarithmized, grouped and filtered for min. valid number (min. 3 in at least one group). Missing values have been replaced by random numbers that are drawn from a normal distribution. Two sample *t*-test was performed using FDR = 0.05. Probability values (p) <0.05 were considered statistically significant. To identify significant enriched GO terms, we utilized the 1D enrichment tool in Perseus. Data visualization was done in the statistical environment R. MS analyses of three independent samples for each experiment were performed. Peptides with Log_2_ Difference ≥1 and -Log *p*-value > 1.3 were considered significantly enriched.

## Supplementary information

Figure S1

Figure S2

Figure S3

Figure S4

Supplementary Figure Legends

Original Data

## Data Availability

RNA sequencing and ChIP-sequencing data have been deposited on GEO (GSE260617 and GSE260620, respectively). Proteomic data are available via ProteomeXchange with identifier PXD052342.
