# Supplementary figures and images for: TFEB controls syncytiotrophoblast formation and hormone production in placenta
Source: Cell Death Differ. 2024 Jul 4;31(11):1439–51. doi: 10.1038/s41418-024-01337-y (PMC11519894; doi:10.1038/s41418-024-01337-y)

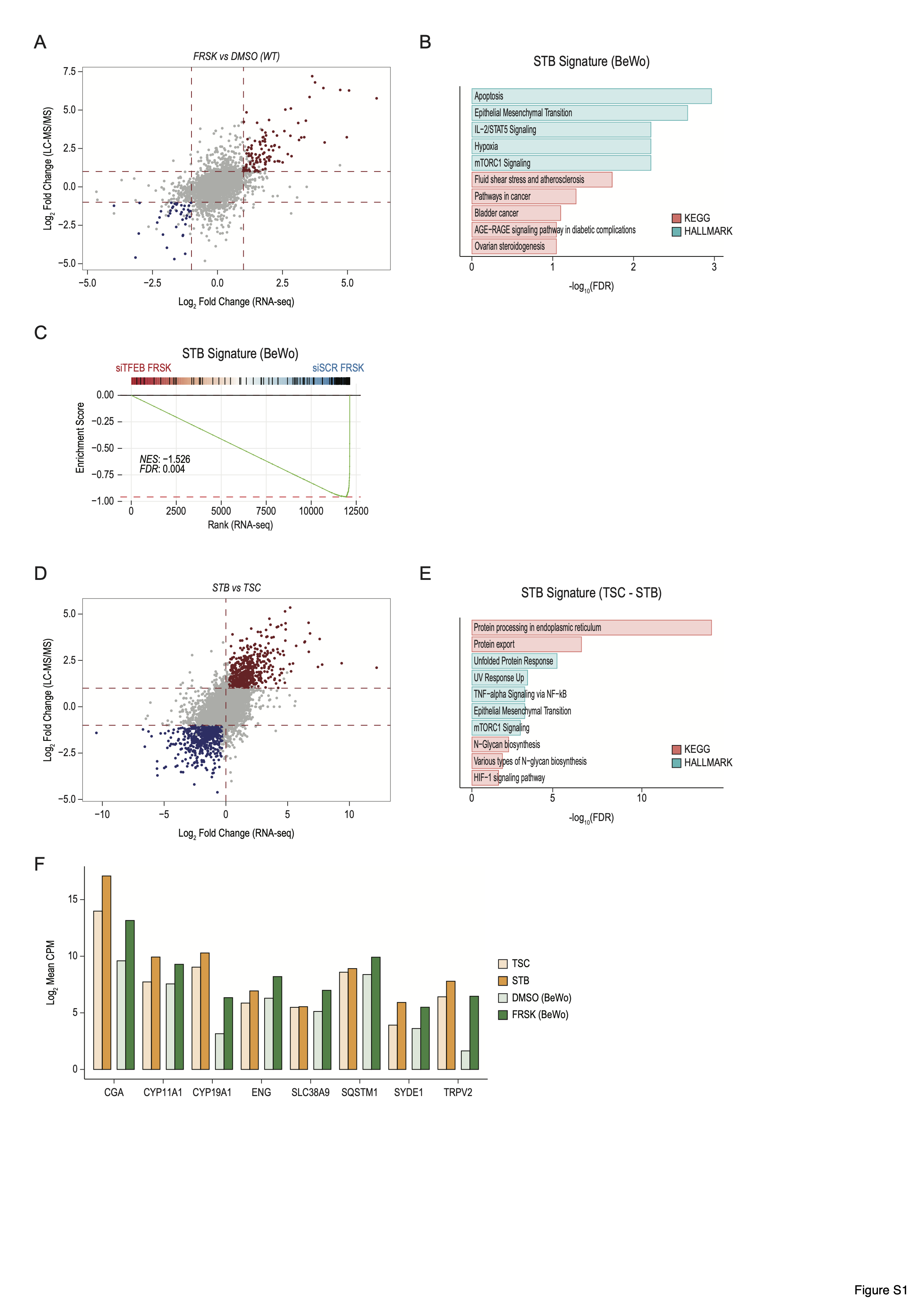

Supplement: Supplementary file 1 — Figure S1 [file 41418_2024_1337_MOESM1_ESM.png]

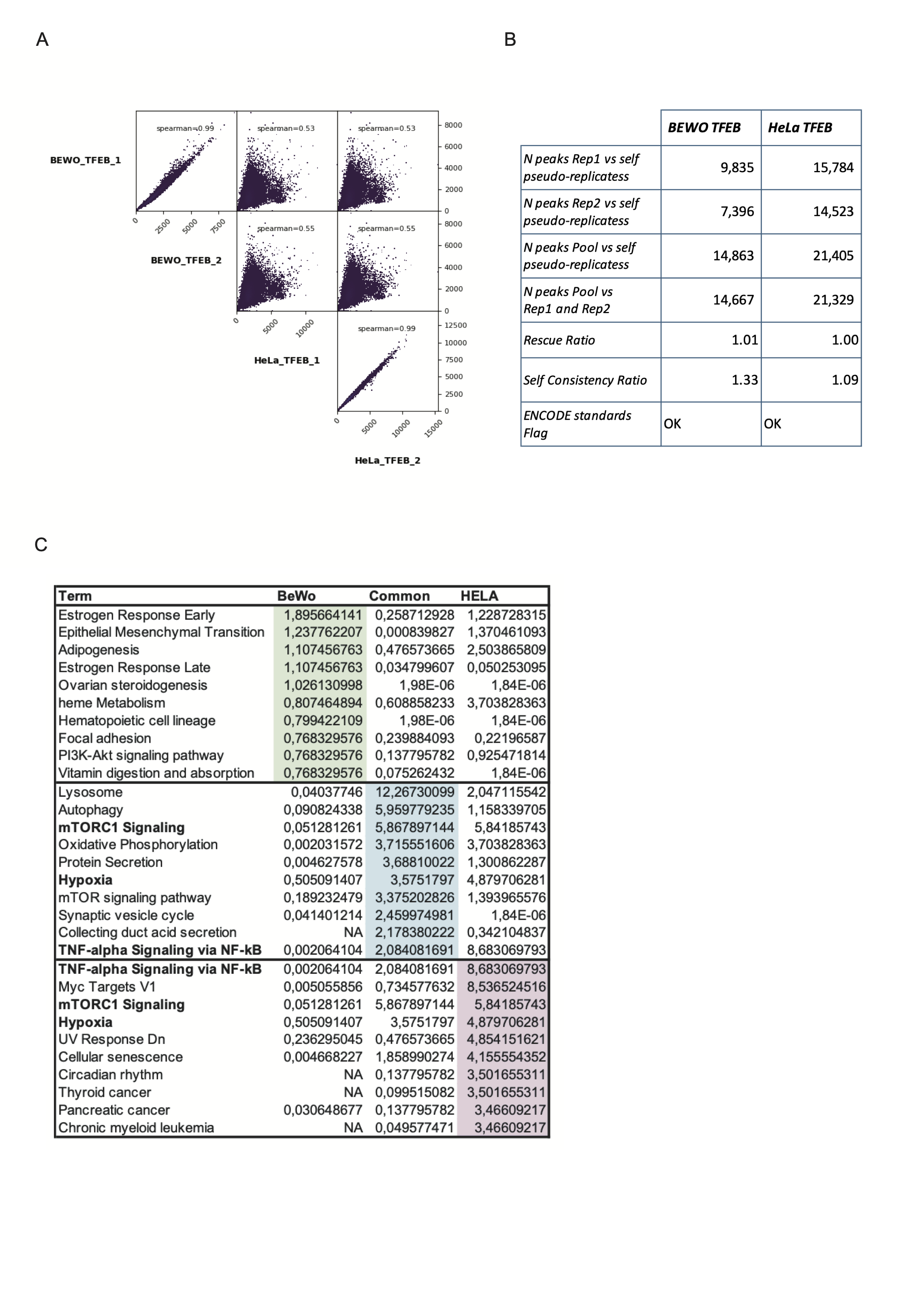

Supplement: Supplementary file 2 — Figure S2 [file 41418_2024_1337_MOESM2_ESM.png]

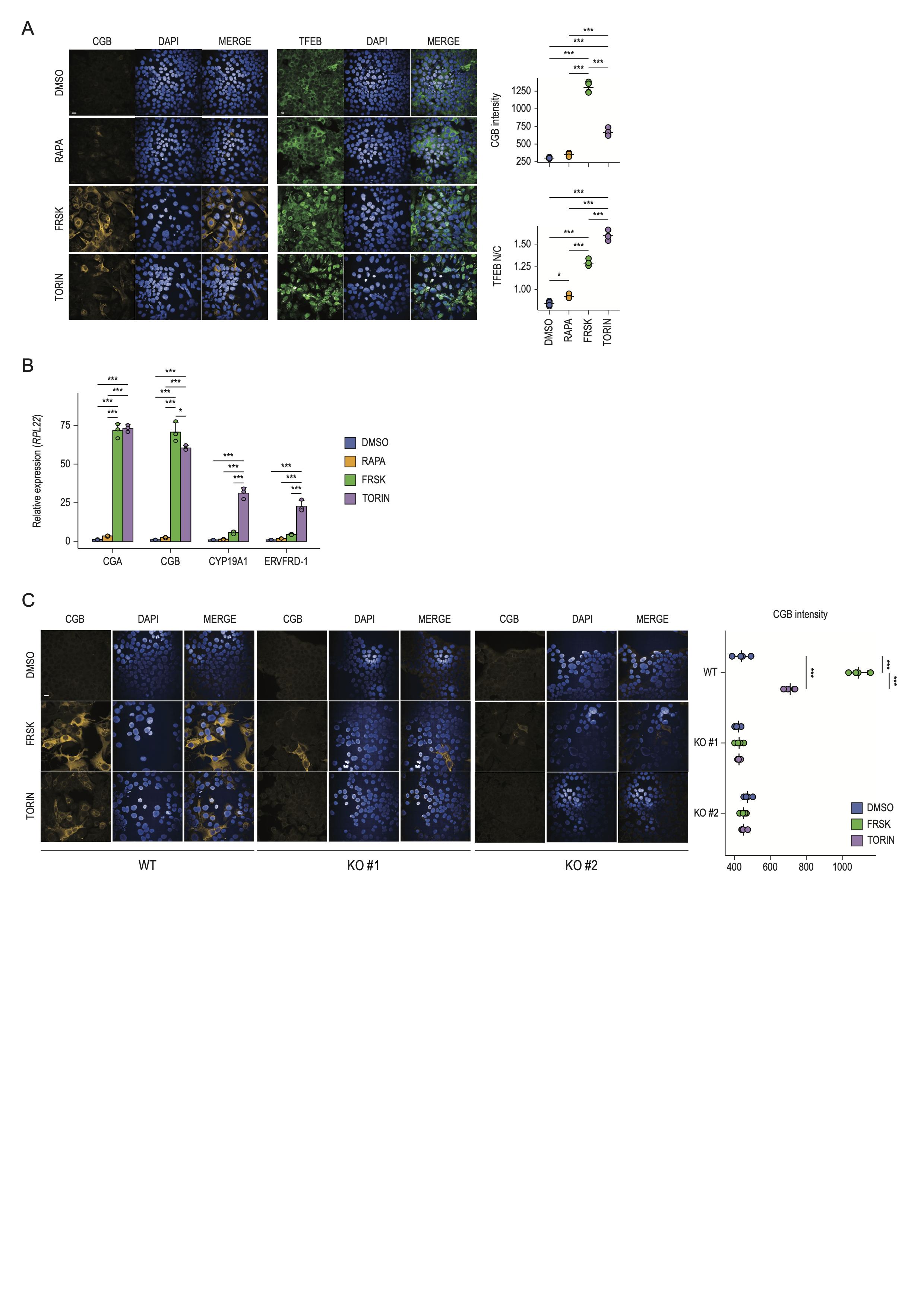

Supplement: Supplementary file 3 — Figure S3 [file 41418_2024_1337_MOESM3_ESM.png]

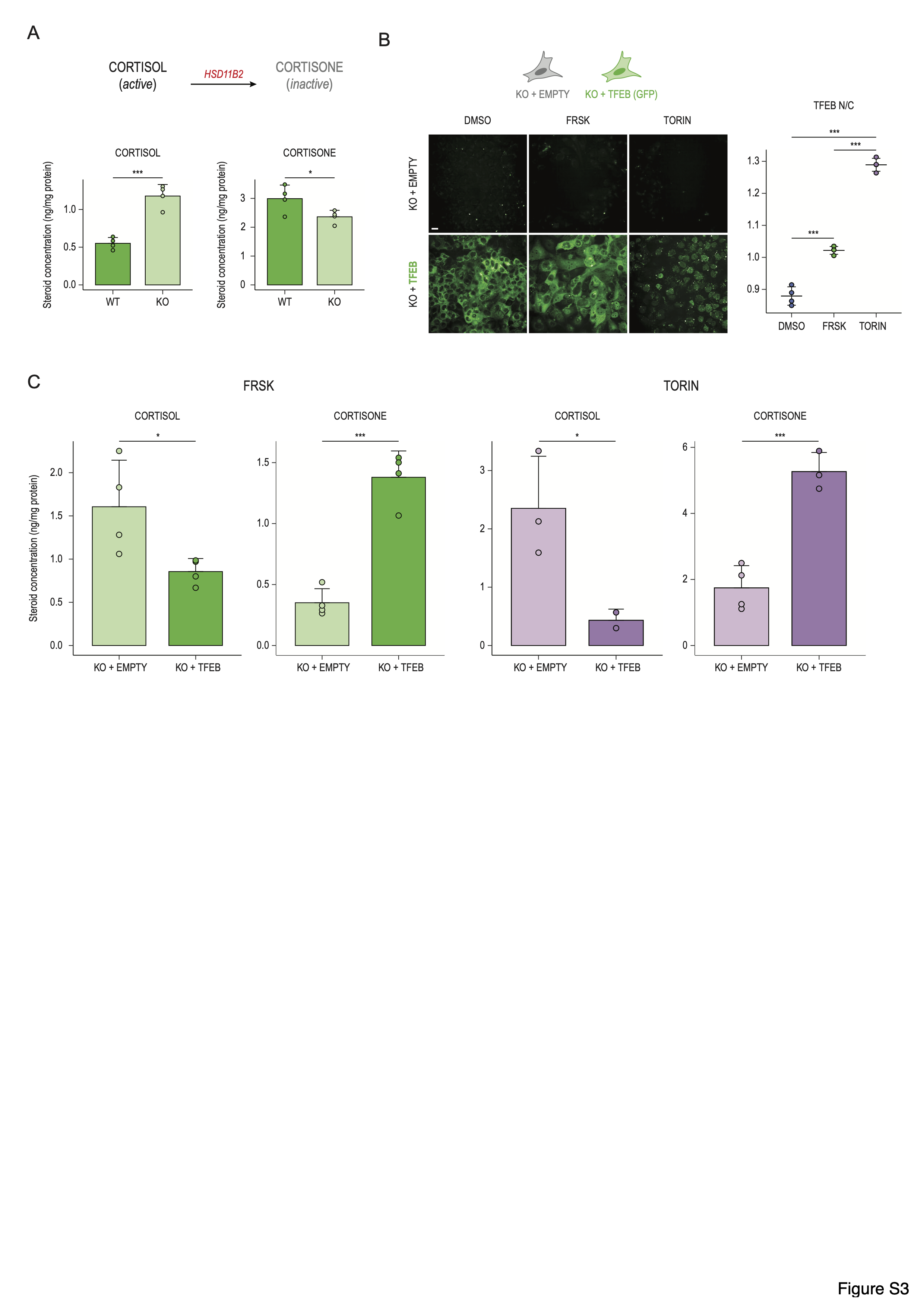

Supplement: Supplementary file 4 — Figure S4 [file 41418_2024_1337_MOESM4_ESM.png]

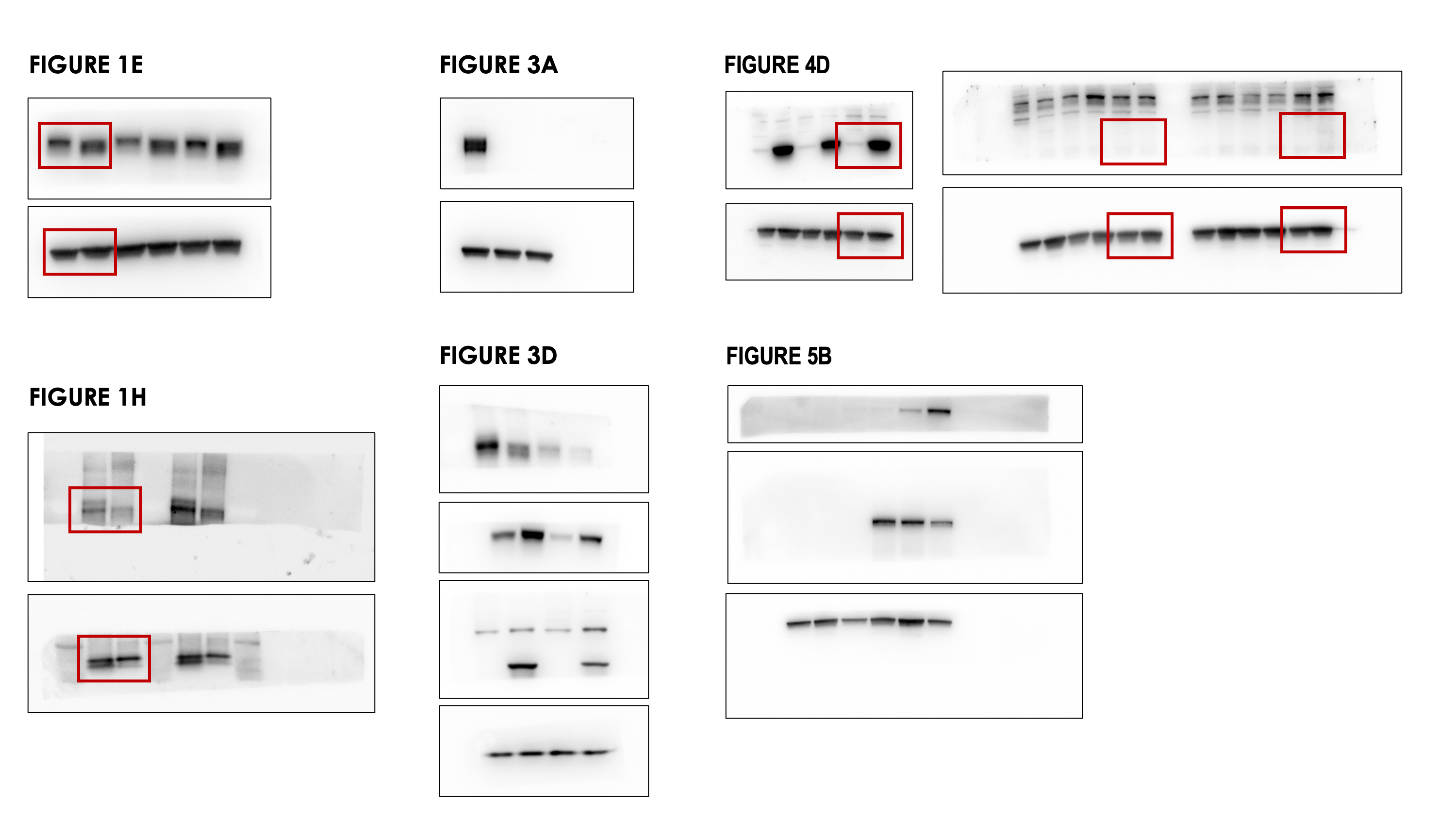

Supplement: Supplementary file 6 — Original Data [file 41418_2024_1337_MOESM6_ESM.png]
