# Supplementary material for: TFEB controls syncytiotrophoblast formation and hormone production in placenta
Source: Cell Death Differ. 2024 Jul 4;31(11):1439–51. doi: 10.1038/s41418-024-01337-y (PMC11519894; doi:10.1038/s41418-024-01337-y)
Supplement: Supplementary file 5 — Supplementary Figure Legends [file 41418_2024_1337_MOESM5_ESM.docx]

**SUPPLEMENTARY INFORMATION**

**Figure S1. Definition of gene signatures in BeWo cells and IPSC-derived TSCs**

**A:** Volcano plot of genes expressed in BeWo cells upon DMSO and Forskolin (FRSK) treatments and displayed as Log_2_ Fold Change in RNAseq (x-axis) and LC-MS/MS (y-axis). Genes significantly upregulated in FRSK versus DMSO at both RNA and protein levels are represented as red dots. Genes significantly downregulated in FRSK versus DMSO at both RNA and protein levels are represented as blue dots.

**B:** Bar plot of representative term enrichment analysis results using Curated Pathways (KEGG and MSigDB Hallmark collection) of genes belonging to the “STB signature - BeWo”, consisting of genes upregulated at both RNA and protein levels in BeWo cells upon Forskolin (FRSK) treatment compared to DMSO.

**C:** Gene-Set Enrichment Analysis was performed on RNAseq-detected genes ranked by their fold-change and significance (see Methods) in FRSK-treated BeWo cells upon TFEB knock-down (siTFEB) against siRNA control (siSCR). Upregulated genes from panel S1A (STB signature - BeWo) were used as geneset. Normalized Enrichment Score (NES) and False Discovery Rate (FDR) are reported.

**D:** Volcano plot of genes expressed in human naive IPSC-derived trophoblast stem cells (TSC) and differentiated syncytiotrophoblast (STB) and displayed as Log_2_ Fold Change in RNAseq (x-axis) and LC-MS/MS (y-axis). Genes significantly upregulated in STB versus TSC at both RNA and protein levels are represented as red dots. Genes significantly downregulated in STB versus TSC at both RNA and protein levels are represented as blue dots.

**E:** Bar plot of representative term enrichment analysis results using Curated Pathways (KEGG and MSigDB Hallmark collection) of genes belonging to the “STB signature (TSC-STB)”, consisting of genes upregulated at both RNA and protein levels in STB cells compared to TSC.

**F:** Bar plot of representative genes common to both STB signatures, shown as averaged log_2_ normalized expression (CPM).

**Figure S2. Extended ChIPseq results**

**A:** Spearman correlation of individual ChiPseq replicates.

**B:** Replicate concordance in ChIP-seq experiments was measured by calculating IDR values (Irreproducible Discovery Rate). According to ENCODE standards, having both rescue ratio and self consistency ratio values < 2 is recommended, but having only one of the ratio values < 2 is acceptable.

**C:** Top-10 terms enriched for each group of TFEB targets (BeWo, Common and HeLa -specific) displayed as of -Log10(FDR). In bold are indicated terms common to more than one group.

**Figure S3. Functional assays of syncyzialization in BeWo cells by using High Content Imaging**

**A:** (*Left*) Representative immunofluorescence images of BeWo cells upon DMSO, Rapamycin (RAPA), Forskolin (FRSK) and Torin-1 (TORIN) treatments immunostained for human chorion gonadotropin β subunit (CGB) and TFEB. Nuclei are counterstained blue using DAPI. Scale bar 20 µm. (*Right*) High-content imaging-based quantification of CGB intensity and TFEB fluorescence intensity in the nucleus and the cytoplasm (TFEB N/C) upon indicated treatments (n=4) are shown as dot plots. Statistical analysis was performed by One-way analysis of variance (ANOVA) followed by Tukey's multiple comparisons test (*, p ≤ 0.05; **, p ≤ 0.01; ***, p ≤ 0.001).

**B:** Bar plots showing relative CGA, CGB, CYP19A1 and ERVFRD-1 mRNA levels measured by qPCR in BeWo cells treated with DMSO, Rapamycin (RAPA), Forskolin (FRSK) and Torin-1 (TORIN). Values were normalized on the RPL22 expression and displayed as a fold change relative to DMSO conditions set to 1 (n=3). Mean ± SD values are shown. Statistical analysis was performed by One-way analysis of variance (ANOVA) followed by Tukey's multiple comparisons test (*, p ≤ 0.05; **, p ≤ 0.01; ***, p ≤ 0.001).

**C:** Representative immunofluorescence images of wild-type (WT) and TFEB KO (KO#1, KO#2) cells upon DMSO, Forskolin (FRSK) and Torin-1 (TORIN) treatments immunostained for human chorion gonadotropin β subunit (CGB). Nuclei are counterstained blue using DAPI. Scale bar 20 µm. (*Right*) High-content imaging-based quantification of CGB intensity upon the indicated treatments (n=4) is shown as a dot plot. Statistical analysis was performed by One-way analysis of variance (ANOVA) followed by Tukey's multiple comparisons test (*, p ≤ 0.05; **, p ≤ 0.01; ***, p ≤ 0.001).

**Figure S4. Quantification of cortisol and cortisone in BeWo cells upon STB formation**

**A:**  Bar chart graph representations of the quantitative determination of Cortisol and Cortisone in BeWo wild-type (WT) and TFEB knock-out (KO) cells upon Forskolin (FRSK) treatment detected by UHPLC-MS/MS-based targeted steroidomics. Steroid concentration values are expressed as ng/mg of protein. Statistical analysis was performed by one-sided unpaired Welch’s t-test (*, p ≤ 0.05; **, p ≤ 0.01; ***, p ≤ 0.001).

**B: (***Left*) Representative immunofluorescence images of TFEB knock-out (KO) cells transduced with an empty control vector (KO+EMPTY) or TFEB-GFP overexpressing vector (KO+TFEB) upon treatment with DMSO, Forskolin (FRSK) and Torin-1 (TORIN). TFEB is tagged with a GFP protein to monitor its expression. Scale bar 20 µm. (*Right*) Dot plot showing the ratio relative to the quantification of TFEB fluorescence intensity in the nucleus and the cytoplasm (TFEB N/C) upon indicated treatments (n=4) measured by High-Content Imaging. Statistical analysis was performed by One-way analysis of variance (ANOVA) followed by Tukey's multiple comparisons test (*, p ≤ 0.05; **, p ≤ 0.01; ***, p ≤ 0.001)

**C:**  Bar chart graph representations of the quantitative determination of Cortisol and Cortisone in TFEB knock-out cells transduced with an empty control vector (KO+EMPTY) or TFEB overexpressing vector (KO + TFEB) upon treatment with Forskolin (FRSK) and Torin-1 (TORIN) detected by UHPLC-MS/MS-based targeted steroidomics. Steroid concentration values are expressed as ng/mg of protein. Statistical analysis was performed by one-sided unpaired Welch’s t-test (*, p ≤ 0.05; **, p ≤ 0.01; ***, p ≤ 0.001).
